# Colony Defense Behavior of the Primitively Eusocial Wasp, *Mischocyttarus cerberus* is Related to Age

**DOI:** 10.1673/031.010.13601

**Published:** 2010-08-18

**Authors:** Olga Coutinho Togni, Edilberto Giannotti

**Affiliations:** Departamento de Zoologia - lnstituto de Biociências, Universidade Estadual Paulista, Caixa Postal 199; 13506-900, Rio Claro, SP, BRAZIL

**Keywords:** ethology, hierarchical position, age polyethism

## Abstract

The colony defense behavior of the wasp *Mischocyttarus cerberus* Richards (Hymenoptera, Vespidae) was studied to verify whether there were different reactions of wasps of different ages and hierarchical positions during attacks of ants. Detailed nest mapping was first performed, then the wasps were marked and were divided in four distinct categories: queens, older workers, younger workers and males. Tests were made simulating attacks of ants in the nests. The main results showed that the *M. cerberus* behaviors against ant attacks is more related to the age of the wasps than to their hierarchical position. The oldest wasps (queens and older workers) defend the nest more than the younger workers and males, representing a form of temporal polyethism.

## Introduction

The division of labor of the primitively eusocial wasps, such as *Mischocyttarus cerberus*, is characterized by a beneficial behavioral plasticity, and any wasp, regardless of age or hierarchical position, performs a wide array of tasks in the colony (Giannotti 1999). On the other hand, in *Polistes lanio* it was observed that the dominant females remain longer in the nest than the subordinates (Giannotti and Machado 1999). In *P. chinensis antennalis*, Kasuya (1983) also noted that the workers have a greater number of behaviors outside the nest than the queen. Gadagkar and Joshi (1983) demonstrated the presence of three different behavioral castes, denominated “sitters”, “fighters” and “foragers”. This division of tasks can be related the age of the wasps, and despite temporal polyethism being observed mainly in highly social species of wasps, or species with large colonies and morphological differences between castes, they can also occur in primitive eusocial species (Naug and Gadagkar 1998). In the primitively eusocial bumble bee, *Bombus griseocollis*, differences of behavioral repertory related with age was observed mainly in the initial cohort, however all tasks were performed at all ages (Cameron 1989). Shorter and Tibbetts (2009) demonstrated that older workers of primitively eusocial wasp *Polistes dominulus* spend more time foraging than younger workers and the juvenile hormone mediates this temporal polyethism.

In study with *Apis mellifera*, Breed et al. (1990) concluded that colony defense involves at least two groups of behaviorally and genetically differentiated bees, soldiers and guards. Guards are generally younger than foragers or soldiers. At least some guards become foragers and others become soldiers, specialized defenders. Unlike the honey bee, in the swarm-founding epiponine wasp, *Polybia occidentalis*, the defense against vertebrates is clearly an age polyethism case that is a task performed by older workers (Jeanne et al. 1992). In the same species of wasp, London and Jeanne (2003) conclude that colony-level defensive effort increased as colonies developed through the pre-emergence stage and the intensity of defense is a function of the colony's investment in brood. Previous studies with *M. cerberus* colonies in the stage of pre-emergence showed that the solitary foundress defend the nest only after the appearance of the first larvae (Togni and Giannotti 2007). However, in post-emergent colonies the number of immatures (eggs, larvae, pupae) present represent an important factor in the type and the intensity of defense against attack by ants (Togni and Giannotti 2008).

According to the information above and knowing that the ants are seen as natural enemies of social wasps (Jeanne 1975) and that in *M. cerberus*, alarm behavior is most often performed by the queen rather than by than the workers (Giannotti 1999), the main objective of this study was to determine whether there is difference in behavioral acts of wasps of different age and dominance status of *M. cerberus* during nest defense against attack by ants. Moreover, observations were made on which defense strategies were used, including the use of chemical defense where repellent secretions produced by exocrine glands are deposited by “rubbing” and other similar behaviors, or by the use of active behavior and attack, using the jaws and sting for example.

## Materials and Methods

### Methods of observations

The study was conducted on the campus of UNESP in Rio Claro, São Paulo, Brazil, using a population of *M. cerberus* wasp nests located on the exterior of the building of the Department of Mathematics. In the period from January to June 2006, seven colonies and 43 individuals were subjected to periodic stimulations, totalling 44 hours of field work.

At the onset of the experiment, the nests studied were mapped to verify the number of cells, immatures (eggs, larvae and pupae) and adults, and to verify the colony stage of development. Next, to identify the behavioral roles of each individual (queens, workers and males) in the colony during defense against an attack of ants, the wasps were collected, marked and then returned to the nest. The mark was made on the mesosoma with airplane modelling paint, using a code with five colours and up to two points making it possible to differentially mark up to 55 individuals. As each nest contains, on average, less than 10 wasps, this method is sufficient and allows easy visualization.

Identification of the hierarchy of individuals was made by observations and records of wasp behavior under typical conditions, without any external stimulus. Utilizing the behavioral patterns of *M. cerberus* described by Giannotti (1999), it was possible to identify whether the wasps were queens or workers. Moreover, as the younger wasps of this species have dark black eyes for about a week post-emergence, was possible to separate the younger workers from the older ones. Males were easily identified because the tips of the antennae are bent and the clypeus lighter than females. Thus, wasps were classified according to the following criteria: queen (Q), older workers (OW), younger workers (YO) and male (M). The paint marking, in addition to enabling the identification of each wasp's position in the dominance hierarchy, allowed for the continued identification of individuals throughout the development of the colony.

After the identification of the behavioral roles of wasps in the colonies, bioassays were made, simulating ants attacks on nests. As a control, tweezers cleaned with alcohol were used, and held near the nest for a minute to observe the reaction of wasps to the foreign object. A second stimulation was made after the wasps had calmed down. This time, another tweezers holding a worker of the ant *Camponotus crassus* Mayr (Hymenoptera: Formicidae) was kept near the nest of *M. cerberus*, also for a minute. The live ant was held in tweezers by mesosomum so that the legs were free for the movement. In both situations control and attack of ants, the tweezers were maintained immobile at about a centimeter of distance, within reach of the wasps.

### Recording data

The behavior of each individual, previously marked, was recorded in accordance with its hierarchical position in the colony, making it possible to verify whether there was a difference in behavior among queens, older workers, younger ones and males during the simulated attack by an ant. The colonies evaluated were all at the stage of post-emergence and with more than one individual in the nest, since those are the conditions that make it possible to compare the behaviors of different castes and individuals of the colony.

The behaviors of the post-emergent wasps were recorded by a camcorder to allow the analysis and comparison with the other nests. Further, using TV sets and VCRs, the behaviors of the individuals in the control situation and in the nest attack simulation were observed and quantified, taking in account the postures assumed.

### Behavioral repertory

The behavioral repertory observed in the present study is described below:

1. Abdomen pumping: a longitudinal movement of the abdomen, stretching and contracting it over and over. Jeanne (1981) concluded that wasp venom can be used as an alarm pheromone. Thus, pumping the abdomen can be thought as a way of spreading venom into the air, so as to warn the colony. This behavior was frequently observed concurrently with “wing-buzzing”, described below. Abdomen pumping may the homologous to the “abdomen bending” observed in alarm reaction of *M. drewseni* colonies (Jeanne 1972).
2. Biting: a fast movement of the wasp toward the object of stimulus (the tweezers or the ant), closing its mandibles on the object;
3. Checking cells: walking over the nest surface inserting the head into cells and touching them with the antennae;
4. Fly out: the wasp becomes restless because of the stimulus, flies from the nest and then returns rapidly. This behavior had already been described by Post et al. (1988) in *Polistes fuscatus variatus;*
5. Gaster beating: a movement of the gaster against the nest, lifting and lowering the abdomen several times and beating their gaster tips on the nest surface. *Ropalidia opifex* displays a characteristic alarm behavior in which individuals beat their gaster on the leaf on which the nest was constructed (Fortunato et al. 2004). This may be another way of dispersing alarm pheromone, made by the venom gland (Downing 1991), but leaving it impregnated on the nest. Another possibility is that they are spreading the secretion of the VI abdominal sternite glands, an ant repellent; however this behavior is typically done by rubbing the gaster laterally (as described below);
6. Lifting wings: in response to the stimulus, the wasp lifts its wings, adopting alert posture (West-Eberhard 1969);
7. Mandible opening and closing: the mandible opens and closes several times. Downing (1991) reported that the secretion of the ectal mandible glands are employed for defending territory, hence, the behavior of opening and closing the mandibles are believed to be a way of disseminating the secretion of these glands;
8. Restless walking: the wasp walks on the nest in circles and/or zigzags. This may the homologous to the behavior termed “aggressively darting” observed by West-Eberhard (1969) in *Polistes fuscatus;*
9. Rubbing: a lateral movement of the gaster against the nest; the wasp moves its abdomen in contact with the nest from one side to the other several times. This behavior, reported initially by Jeanne (1970, 1972), is represented by the action of the females applying ant-repellent secretion by rubbing the tuft of hair on the terminal gastral sternum against the nest. This behavior has been observed in the wasp genera that have independent foundation, such as *Mischocyttarus, Polistes, Parapolybia, Ropalidia* and *Belonogaster;*
10. Wing buzzing: in this restless behavior, the wasp vibrates its wings, as though it were going to fly, with short time intervals of about one second;
11. Wing buzzing constantly: in this restless behavior, with no time intervals, the wasp vibrates its wings as though it were going to fly. This behavior and the behavior described above, despite being a clear signal of defensiveness, may also be a way of dispersing alarm pheromone (venom), as it is often exhibited together with the abdomen pumping behavior (Jeanne 1972). This may the homologous to the “wing-flipping” observed in *Polistes fuscatus* (West-Eberhard 1969);
12. Abandoning the nest: the wasp reacts to the stimulus by leaving the nest and flying away;
13. Hiding: the wasp goes to the back of the nest and remains immobile;
14. Ignoring: in this behavior the wasp does not react to the stimulus at all, and keeps its normal behavior in the nest, performing nest maintenance tasks normally;
15. Immobile: during the experiment the wasp reacted by not moving, apparently inactive on the nest;
16. Peaceful walking: the wasp walks calmly and slowly through the nest;
17. Touching with the antennae: the wasp puts its antennae in contact with the object (the ant or the tweezers);
18. Touching with the legs: the wasp put their front legs in contact with the object;
19. Arriving: a wasp that was absent from the nest throughout the experiment arrives at the nest;
20. Dominance behavior: attack behavior of a wasp towards another with a lower hierarchical level by lifting up the front legs and antennae and biting the other female. Other behaviors related to social dominance, as oophagy and trophallaxis adult-adult, have been described for *M. cerberus* (Giannotti 1999), however these were not observed in this study;
21. Fly around the nest: a wasp flies in the vicinity of the nest;
22. Returning: a wasp that abandoned the nest during the experiment then returns to the nest;
23. Self-grooming: a wasp rubs its legs together or rubs them on its mandibles and/or antennae, cleaning itself;
24. Submissive behavior: when the subordinate wasp receives dominative behavior from another female;

This repertoire, totalling 24 behavioral acts, was divided into 3 separate groups for better viewing and understanding of the experiment. The former group includes those considered aggressive behavior and/or more intense to defend the nest, named “Defensive” (behaviors 1 to 11). The second group included behaviors considered less intense or not defensive and were named as “Nondefensive” (behaviors 12 to 18). The third group was created to include behaviors that do not fit into either of the two previous groups, which was called “Others”(behaviors 19 to 24), because it contains behaviors considered independent of the stimuli offered.

### Statistical methods

The statistics analysis was employed using the software STATISTICA 8.0. At first, the data obtained were analyzed using the Mann-Whitney U test, verifying differences between the sum of frequency of behaviors accomplished of older wasps (queens and older workers) and young wasps (younger workers and males which most often are also younger, as they remain for a short time in the colony).

**Figure 1.**
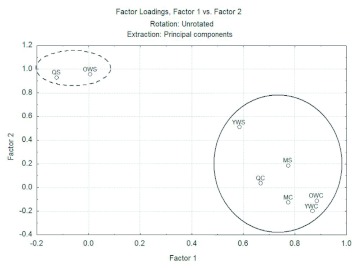
Behavioral repertory of *Mischocyttarus cerberus* in the control situation and in the simulated attacks by ants analyzed together with all individuals data according hierarchical position and two principal components. Individuals that are inside dot line circle (queens and older workers during simulation of attack by ants) reacted with more defensiveness than the others individuals that are inside solid line circle. High quality figures are available online.

**Figure 2.**
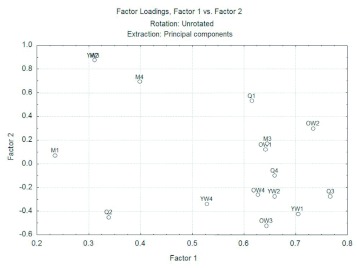
Behavioral repertory of *Mischocyttarus cerberus* in the control situation analyzing the wasps individually according there age and hierarchical position and two principal components. High quality figures are available online.

**Figure 3.**
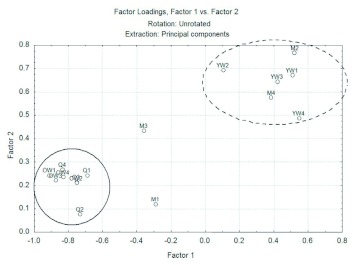
Principal Component Analysis of behavioral repertory of *Mischocyttarus cerberus* in the simulated attacks by ants according the wasps individually and there age and hierarchical position. Individuals that are inside solid line circle (queens and older workers) reacted with more defensiveness than the others individuals that are inside dot line circle (younger workers and males). High quality figures are available online.

For a more detailed multivariate analysis, Principal Component Analysis (PCA) was used that transforms to a linear representation a large group of variables into another group with fewer and non-correlated variables. PCA was used initially to analyze the female groups and males in control situations and simulation of ant attack (Figure 1). Next, for a better use and visualization of the analysis, 4 individuals were selected from each male and females groups, being those that presented the largest number of behaviors and data collected. These selected individuals were analyzed separately in each treatment, control situation and simulation of ant attack, explaining the reaction of each one of those individuals in these situations (Figure 2–3).

The nonparametric Fisher test was used to complement the analysis and compare the frequencies of each behavior performed by the older and younger wasps in the control situation and in simulation of ant attack. The results are included in Table 1 and help distinguish which behaviors are most important.

## Results

First the behavioral data obtained were summarized in Table 1, which presents the frequency (%) of the behaviors associated with each group of wasps during the control situation and the simulated ant attack.

In the control situation, although the queens and workers had taken some defensive behaviors, the behavioral acts of immobile and ignoring were also considerably frequent. In general, all subjects showed some defensive behavior and, probably because of that, the total behavior repertoire of the younger wasps and older wasps did not differ significantly according Mann-Whitney U test (z = 0.8998; p = 0.1841). But it can be observed (Table 1) that the queens and the older workers were more defensive at the time of recognition of the foreign object (tweezers), than the younger workers and males, since the latter did not display any behavior considered defensive in high percentage. Moreover, in the last column of Table 1 (Fisher's test result) it can be observed that the behavior “touching with the antennae” (p = 0.0419) in the control situation was significantly more frequent than in the simulation of ant attack, showing that wasps reacted to the tweezers less aggressively than the ants.

During the simulated ant attack, both the queen and the older workers, after realizing the presence of the enemy, touched the ant with the antenna, lifted the wings, performed wing buzzing, bit the ant and pumped the abdomen several times. Some older workers and queens beat, rubbed their gasters on the nest and flew out and these behaviors are important because they were performed only by oldest wasps showing that oldest individuals more often use chemical defensive behaviors than younger individuals Other queens did restless walking and dominating their subordinates. Some older workers did wing buzzing and wing buzzing constantly, and this behavior was only observed in the simulation of an ant attack Unlike the oldest individuals, the younger workers and males ignored the stimulus, were immobile and abandoned the nest significantly more frequency according the Fisher Test in Table 1 (p = 0.0158; p = 0.0171; p = 0.0396, respectively). In all categories, the act of abandoning the nest was more often observed in males. Although hiding did not differ significantly, this behavior was more often observed in younger workers and males (Table 1). The total behavior repertoire of both groups older individuals (queens and older workers) and younger individuals (younger workers and males) showed significant differences according to the Mann-Whitney U test (z = 1.7033; p = 0.0443).

The data obtained in the control situation and in the simulated attacks by ants were then analyzed together, using the method of PCA, generating a better view. In Figure 1, the acronyms followed by “C” represent the individuals of the females and male groups position in the control situation, while the symbols followed by “S” represent the simulation of an attack of ants, for example, “QC” are the queens during the control situation, the letters “QS”, represent the same queens in the simulation of an attack by ants. It is then possible to observe two distinct groups, the first (dotted line) formed by the queens in the simulation of an attack by ants (QS) and the older workers in the same situation (OWS) at the bottom left of the graph; and the second group (solid line) formed by the others wasps in the two situations (YWS, MS, QC, OWC, YWC, MC). Thus we can conclude that the queens and the older workers react with more defensiveness during the simulation of an attack of ants than the others, since they are more related to defensive behaviors that define the second principal component. This strengthens the possibility that nest protection against ants is closely related to the age of wasps and not to the different groups of adults. The fact that this difference did not occur in the control situation and that all groups of wasps did not show defensive behavior (they are more correlated with the first principal component), as well as the males and younger workers in the simulation of attack by ants, highlights the difference in wasps between the control and simulation of attack by ants, demonstrating the efficiency of the method. More information about this Principal Component Analysis is in Supplement 1 (available online).

**Table 1.**
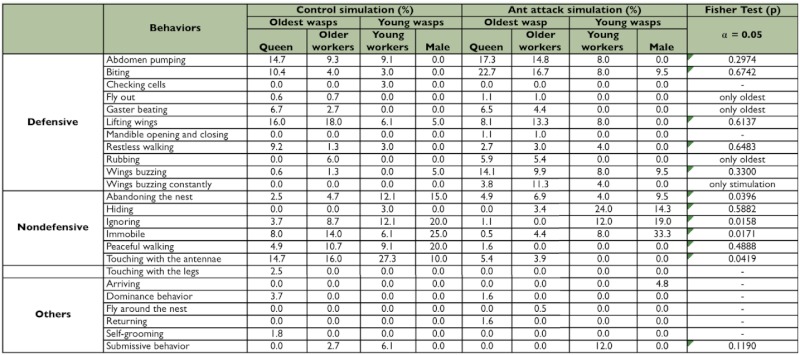
Comparing each behavior using the Fisher test (α = 0.05) and their frequency (percentage) accomplished by each group of individuals, queens and older workers (oldest wasps) and young workers and males (also young wasps) in the control situation and in the simulation of attack by ants.

PCA helped elucidate differences in the overall behavioural patterns of the four groups, considering the individual position of wasps in the hierarchy of the colony. For better viewing in the analysis, 4 individuals were selected in each male and females groups with more data, and these were numbered 1 to 4 at random.

Figure 2 shows the data for wasps in the control situation. Notice that there is no formation of separate groups, individuals are distributed randomly within the graph area. This random distribution in Figure 2 during control stimulation demonstrates that wasps of the colony did not react defensively in contact with a foreign object, since the tweezers was not seen as a threat. More information about this analysis is in the Supplement 2 (available online).Figure 3 contains the data for *M. cerberus* for the simulation of an attack by ants. Observe the formation of two separate groups at the time of nest protection against ants. The first group (dotted line) is formed by younger workers and by males, which most often are also younger, as they remain for a short time in the colony. This group provides a high correlation with both the first and the second principal component, and therefore have nondefensive behaviors such as remaining immobile, hiding and ignoring, in the top right of the chart. The second group (solid line) consists of queens and older workers, and as it is in the lower left region of the graph it is represented mainly by high negative values of the first major component, such as biting, abdomen pumping and lifting wings, that are all considered defensive behaviors. This information further reinforces the conclusion made previously that nest protection against ants depends on the age of the individuals. In Figure 3, we can see that male 3 (M3), which had yellowish eyes, showing that it was older, had more defensive behaviors approaching the second group. Male 1 (M1), although not having yellowish eyes, flew during the stimulus and, therefore moved from the first group. More information about this analysis is in the Supplement 3 (available online).

## Discussion

This paper provides a detailed investigation of the defensive responses of individuals differing in age and dominance status of the primitively eusocial wasp, *M. cerberus*, and shows that during the control situation, despite the fact that all the females showed the act of abdomen pumping, it was frequently noted that in all groups wasp behaviors was considered nondefensive, such as touching with the antennae, peaceful walking and remaining immobile. Moreover, there was a low percentage of the accumulated variance (Table 3), which probably occurred due to lack of a behavioral pattern observed during the control stimulation, highlighting the effectiveness of the method. These results show that the wasps do not attack any foreign object or individual that approaches the nest, emphasizing the statement proposed by Jeanne (1975) that the ants are natural enemies of wasps and they evolved strategies to defend the nest against attack from these predators such as the “rubbing”.

The rubbing behavior is characteristic of the fact that the wasp impregnates the nest with a secretion produced by the Van der Vecht gland located on the sixth gastral sternite, that is recognized as an ant repellent (Jeanne 1970, 1972). Raposo-Filho and Rodrigues (1987) and Raposo-Filho (1989) observed that the dominant and subordinate females and males of *M. extinctus* have this organ, allowing any individual when emerging to perform this defense of the colony. The ability to produce ant repellent is a key adaptation of independent foundation wasps, with all species studied by Smith et al. (2001) having the Van der Vecht gland and its ant repellent secretion.

Gadagkar (1980) observed in *Ropalidia marginata* that the queens and the dominants perform more alarm reactions than the subordinates. In *M. cerberus*, in addition to the behavior of alarm, the queens also more frequently performed the act of rubbing the gaster (Giannotti 1999). In *Ropalidia fasciata* (Kojima 1984) and *Polistes metricus* (Gamboa et al. 1978) was also observed that queens more frequently performed “rubbing” than the subordinates. Nevertheless, it was concluded in our work that the older workers when they defend their nests react as the queens do (Figure 3). This difference was due to the fact that the workers were divided into two categories, older workers and younger workers.

In this study, it is possible to see that the protection of the nest against ants is related to age of individuals, as the wasps that defended chemically (abdomen pumping, rubbing and gaster beating) as well as actively (biting and stinging) the colony, queens and older workers have been in the nest for more time than the younger workers and males that emerged recently and more frequently showed nondefensive behavior (Table 1). It is interesting to emphasize that the few times that the second group defended the colony it showed only active defense, wings buzzing and biting the ant, not being observed the act of rubbing and gaster beating. Probably, this lack of chemical defense in the younger individuals is due to the fact that in these individuals the glands related to these behaviours are less developed, according to Downing and Jeanne (1983), who observed in *Polistes fuscatus* that in the dominant wasps, exocrine glands, including those on the sixth gastral sternite, are more developed.

Judd (2000) noted that the queens of *Polistes fuscatus* are consistently the most defensive individuals of the colony and that the subordinate foundresses and workers are more defensive against a vertebrate predator in accordance with age. In *Polistes canadensis*, it was found that the behavior of defense including bites or attacks against an individual or a foreign object has a strong relationship with age, as this was observed mainly between the fourth and 11th days of life of the individuals. It was also observed that the behavior of escaping and flying were more frequent in 4 day-old workers, while older wasps more often perform the behavior of guardians of the colony (Giray et al. 2005). In this study, great differences between the behavior of flying in accordance with the hierarchy and age group of individuals were not established, although it was possible to observe that more mature wasps participate in a more intense way in the defense of the colony against ants.

In addtion to the relationship between nest defense against ants and age of individuals, the fact is that the colonies of *M. cerberus* are small and therefore the individual in the nest at the time of the attack needs to defend it regardless of its hierarchical position.

It is also important to remember that males exhibit behavior similar to that of younger workers because they remain for a short time in the nest, and the males of this species have an average longevity of 10 days (Giannotti 1999), and therefore are considered young.

Moreover, males have less relationship with the females of the colony than they have among themselves, so they are less committed to defending the nest than other individuals. While sisters of the colony shared seventy-five per cent of genes in common, males had only twenty-five percent of their genes in common with their sisters, therefore defending the sisters would not be the best way to increase their fitness (Hamilton 1964). In this work, despite the males failure to participate in nest defense against ants, presenting few defensive behaviors, most of the time they ignore the stimulus, hide and remain immobile as has been observed by Togni and Giannotti (2006). In *Polistes lanio* it was observed that under normal conditions, males show a low frequency of the behavior of alarm (4.8%), while most of the time they remain immobile (82.8%) (Giannotti 2004).

In general, two distinct behavioral groups were formed at the time of nest defense against attack of ants, the first formed by the queens and older workers which defend the nest actively and chemically and the second formed by younger workers and males, more frequently demonstrating nondefensive behaviors. This is a form of age polyethism, in which the older wasps more actively defend the colony. Jeanne et al. (1992) had already observed a similar temporal polyethism in *Polybia occidentalis* and they concluded that since the colony defense is a risky task, the strategy of defending the colony performed by older individuals perhaps is a fitness-maximizing strategy of these older workers whose chances of start a new colony are approaching zero.
